# Vitamin D and Intestinal Diseases: Impact on Intestinal Immunity and Gut Barrier Function

**DOI:** 10.3390/diseases14080293

**Published:** 2026-08-13

**Authors:** Erik Shorabaev, Amankeldi Sadanov, Baiken Baimakhanova, Saltanat Orasymbet, Irina Ratnikova, Bakhytzhan Kerimzhanova, Zhanar Assilova, Zaure Datkhayeva, Aknur Turgumbayeva

**Affiliations:** 1 Research and Production Center for Microbiology and Virology LLP, 105 Bogenbay Batyr St., Almaty 050010, Kazakhstan; eshorabaev@mail.ru (E.S.); a.sadanov1951@gmail.com (A.S.); s_orazymbet@inbox.ru (S.O.); iratnikova@list.ru (I.R.); kbf19@mail.ru (B.K.); 2 Women’s Consultation Department, City Polyclinic No. 22, 193A Zhankozha Batyr St., Almaty 050065, Kazakhstan; asilova1986z@mail.ru; 3 Department of General Medical Practice No. 2, Asfendiyarov Kazakh National Medical University, 94 Tole bi, Almaty 050012, Kazakhstan; 4 Faculty of Medicine and Health Care, Al-Farabi Kazakh National University, 71 Al-Farabi Ave., Almaty 050040, Kazakhstan; turgumbayeva.aknur@med-kaznu.com

**Keywords:** vitamin D, vitamin D receptor, intestinal diseases, intestinal barrier, intestinal permeability, gut microbiota, immune system, inflammatory bowel disease

## Abstract

Background/Objectives: Intestinal diseases are a major global health problem due to their high prevalence, chronic course, and significant impact on quality of life. Increasing attention has been given to the extra-skeletal effects of vitamin D, particularly its role in immune regulation, maintenance of intestinal barrier integrity, and modulation of the gut microbiota. The aim of this review was to evaluate current evidence regarding the role of vitamin D in immune regulation, intestinal barrier function, and the pathogenic mechanisms of intestinal diseases, including inflammatory and functional gastrointestinal disorders. Methods: A structured narrative review was conducted using the PubMed, Scopus, and Web of Science databases to identify publications addressing the role of vitamin D in intestinal diseases. The literature search was updated in May 2026 and included studies published between 2004 and 2026. The aim of this review was to summarize current evidence on the effects of vitamin D on immune regulation, intestinal barrier integrity, gut microbiota, and their clinical relevance in intestinal diseases. Results: The included studies demonstrated that vitamin D plays an important role in maintaining intestinal homeostasis through regulation of innate and adaptive immune responses, preservation of epithelial barrier integrity, and modulation of gut microbiota composition. Vitamin D deficiency was associated with impaired VDR-dependent signalling, increased intestinal permeability, dysbiosis, and enhanced pro-inflammatory immune responses, which may contribute to the development and progression of inflammatory bowel disease, Crohn’s disease, ulcerative colitis, irritable bowel syndrome, and celiac disease. Conclusions: Current evidence supports the important role of vitamin D in immune regulation, intestinal barrier protection, and maintenance of gut microbial balance. Further clinical studies are required to better understand its therapeutic potential in intestinal diseases.

## 1. Introduction

Intestinal diseases represent a significant global burden on healthcare systems and encompass a broad spectrum of chronic inflammatory, autoimmune, functional, and infectious disorders, including inflammatory bowel diseases, irritable bowel syndrome (IBS), celiac disease, infectious intestinal diseases, and colorectal cancer [1,2,3,4]. The prevalence of several intestinal disorders, particularly inflammatory bowel diseases and irritable bowel syndrome, remains high or continues to increase, accompanied by a substantial reduction in patients’ quality of life and a considerable socioeconomic burden [4]. Despite differences in etiology, clinical course, and morphological characteristics, many intestinal diseases share common pathophysiological mechanisms, including chronic inflammation, immune dysregulation, impaired intestinal barrier function, increased intestinal permeability, and alterations in gut microbiota composition [5,6,7,8,9]. Collectively, these disturbances contribute not only to local damage of the intestinal mucosa but also to the development of systemic metabolic and immunoinflammatory consequences, highlighting the importance of identifying modifiable biological factors capable of influencing intestinal homeostasis and the course of these diseases [6].

Vitamin D has traditionally been recognized as a key regulator of calcium–phosphorus homeostasis and bone metabolism [10]. However, current evidence has greatly expanded our understanding of its biological functions. Following sequential hydroxylation in the liver and kidneys, vitamin D is converted to its biologically active hormonal form, calcitriol (1,25-dihydroxyvitamin D), which plays an important role in immune regulation, maintenance of epithelial barrier integrity, and other physiological processes beyond mineral metabolism [11]. Most of its biological effects are mediated through the vitamin D receptor (VDR), a nuclear transcription factor widely expressed in different tissues, including innate and adaptive immune cells as well as intestinal epithelial cells [12]. An important feature of vitamin D biology is the ability of several extra-renal tissues, including immune cells, to locally produce its active form, allowing autocrine and paracrine regulation of cellular functions [13]. Therefore, vitamin D is increasingly recognized not only as an endocrine regulator of mineral homeostasis but also as an important immunomodulatory factor involved in the regulation of inflammatory responses, cellular homeostasis, antimicrobial defense, and tissue repair [14].

The role of vitamin D in the gastrointestinal tract is of particular interest, as the intestine serves not only digestive and absorptive functions but also represents one of the largest immunological interfaces in the human body [8]. The intestinal mucosa is continuously exposed to dietary antigens, microorganisms, microbial metabolites, and other luminal factors, requiring a tightly regulated balance between immune tolerance and protective immune responses [9]. A central component of this process is the intestinal barrier, a complex protective system that includes the epithelial layer, tight junctions, the mucus barrier, antimicrobial peptides, mucosal immune cells, and the commensal gut microbiota [5]. Disruption of the structural or functional integrity of this system may lead to increased intestinal permeability, translocation of microbial components and food antigens, activation of inflammatory signaling pathways, and persistence of chronic inflammation [7].

Accumulating evidence suggests that vitamin D plays an important role in maintaining intestinal homeostasis through several interconnected mechanisms [15]. VDR signaling regulates the expression of tight junction proteins, supports the integrity of the mucosal barrier, enhances antimicrobial peptide production, and modulates the activity of innate and adaptive immune cells [12]. In addition, vitamin D is involved in epithelial repair and restoration of the intestinal mucosa after injury [11]. Another important area is its potential role in regulating the composition and function of the gut microbiota. Recent studies suggest a bidirectional relationship between vitamin D/VDR signaling and the gut microbiota: vitamin D may influence microbial balance, while the microbiota may also affect vitamin D metabolism and related signaling pathways [12,14].

Vitamin D deficiency remains a widespread global public health problem affecting different age groups and geographic regions [10]. In temperate regions, vitamin D status exhibits pronounced seasonal variation because endogenous vitamin D synthesis depends on solar ultraviolet B exposure. Serum 25(OH)D concentrations typically decline during winter months and increase during summer, resulting in a seasonal pattern of vitamin D insufficiency in many populations [16,17]. Beyond its well-known effects on bone metabolism, insufficient vitamin D status has increasingly been associated with immune dysregulation, chronic inflammation, impaired epithelial barrier function, and gut dysbiosis, all of which are mechanisms directly involved in intestinal disease pathogenesis [11,12]. The strongest evidence has been reported in inflammatory bowel disease, where vitamin D deficiency has been associated with increased disease activity, impaired mucosal repair, and poorer clinical outcomes. At the same time, the potential role of vitamin D in IBS, celiac disease, and intestinal infections is being actively investigated, although clinical evidence in these areas remains less consistent [15].

Despite significant progress in understanding the immunomodulatory, barrier-protective, and microbiota-related effects of vitamin D, many aspects of its role in intestinal disease pathogenesis remain unclear [18]. Existing studies often focus on individual mechanisms, such as immune regulation, barrier function, microbiota, or clinical outcomes, without fully integrating these processes into a unified pathophysiological framework. Additional complexity arises from the marked heterogeneity of clinical data, including differences in study design, patient populations, disease phenotypes, baseline vitamin D status, and vitamin D supplementation strategies [13,19]. As a result, the causal role of vitamin D in intestinal diseases, as well as its therapeutic potential, remains an active area of scientific discussion.

This review aims to critically summarize current evidence on the role of vitamin D in the pathogenesis of intestinal diseases, with a focus on immune regulation, maintenance of the intestinal barrier, interactions with the gut microbiota, and potential therapeutic implications.

## 2. Methods

A structured narrative literature search was conducted using the PubMed, Scopus, and Web of Science databases to identify relevant publications on the role of vitamin D in intestinal diseases, with particular emphasis on immune regulation, intestinal barrier integrity, gut microbiota interactions, and potential therapeutic implications. The literature search was last updated in May 2026. No restrictions regarding the year of publication were applied. The studies included in this review were published between 2004 and 2026.

The search strategy combined Medical Subject Headings (MeSH), where applicable, with free-text keywords using the Boolean operators AND and OR. The principal search terms included “Vitamin D”, “Vitamin D Receptor”, “intestinal diseases”, “inflammatory bowel disease,” “Crohn’s disease,” “ulcerative colitis,” “irritable bowel syndrome,” “celiac disease”, “intestinal barrier”, “intestinal permeability”, “gut microbiota” and “immune regulation”. Additional combinations of disease-specific and mechanistic keywords were used to ensure comprehensive coverage of the available literature.

Publications retrieved from the three databases were screened for relevance based on their titles and abstracts, followed by full-text evaluation of potentially eligible articles. Duplicate records identified across databases were removed prior to the final selection of publications.

This review included original research articles, randomized and observational clinical studies, systematic reviews, meta-analyses, narrative reviews, clinical practice guidelines, consensus statements, and other peer-reviewed publications relevant to the objectives of this review. Priority was given to studies investigating the effects of vitamin D on intestinal immune regulation, epithelial barrier function, gut microbiota composition, vitamin D receptor (VDR)-mediated signaling pathways, and clinical outcomes associated with vitamin D deficiency or supplementation. Experimental in vitro and animal studies were considered when they provided mechanistic insights applicable to human intestinal physiology and disease. Where multiple publications addressed similar topics, greater emphasis was placed on higher levels of evidence, including systematic reviews, meta-analyses, randomized controlled trials, large observational studies, and international clinical guidelines.

Publications not written in English, duplicate records, conference abstracts, editorials, letters to the editor, and studies that were not directly relevant to the scope of this review or did not provide sufficient information to address its objectives were excluded.

## 3. Vitamin D Biology: Sources, Metabolism, and Biological Functions

Vitamin D is obtained through endogenous cutaneous synthesis induced by ultraviolet B (UVB) radiation and from exogenous sources, including diet, fortified foods, dietary supplements, and pharmaceutical preparations. Fortified foods and supplements contribute to maintaining adequate vitamin D status, particularly when dietary intake is insufficient [20,21,22,23]. Dietary sources provide only a minor proportion of total vitamin D requirements, whereas endogenous cutaneous synthesis represents the principal physiological source. The efficiency of this process depends on UVB exposure and individual factors affecting cutaneous vitamin D production [24,25,26]. The principal forms of vitamin D are cholecalciferol (vitamin D_3_), synthesized in the skin and found predominantly in foods of animal origin, and ergocalciferol (vitamin D_2_), which is primarily obtained from plant sources and fortified foods [27,28,29].

**Figure 1:**
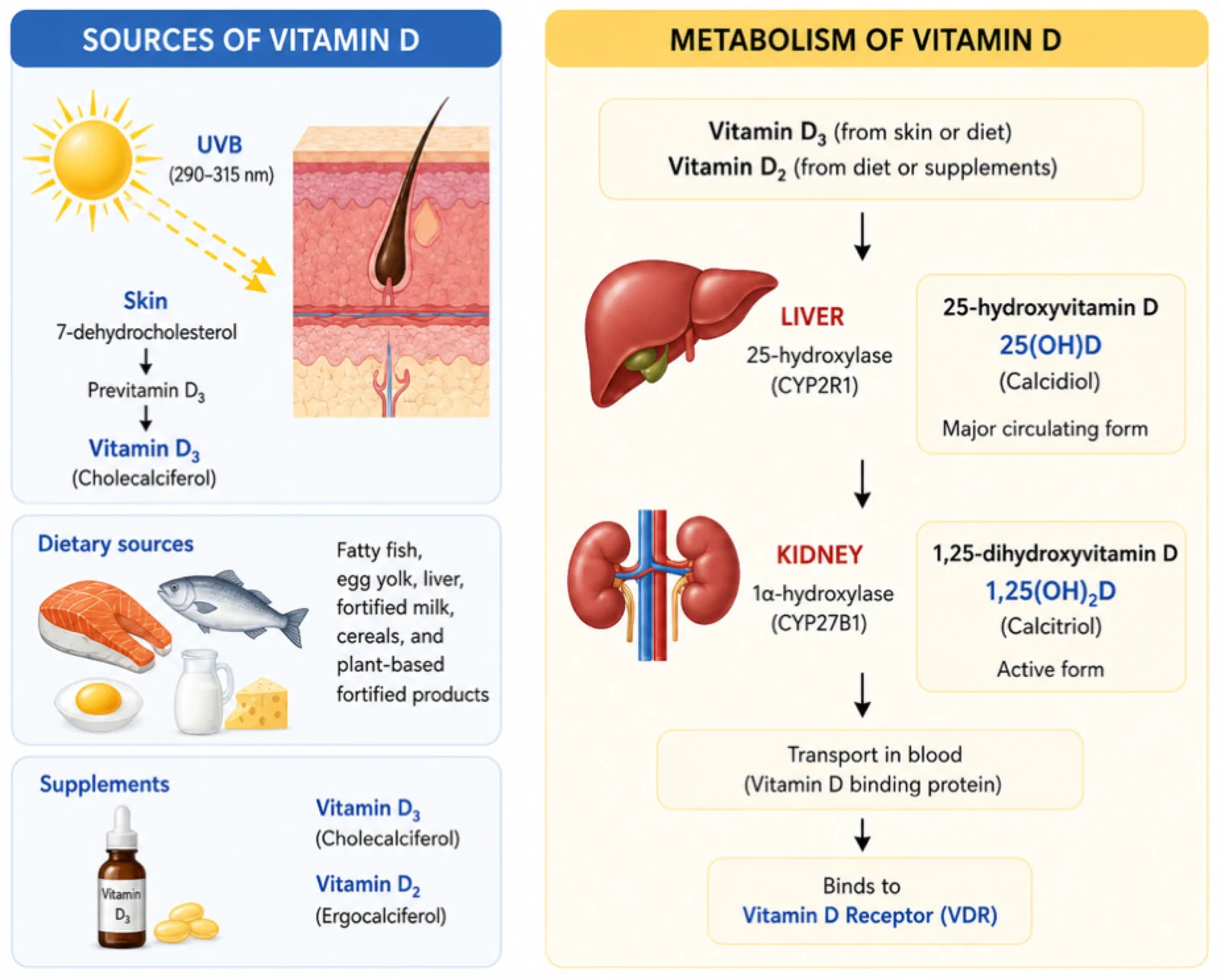
Metabolic activation and VDR-mediated signaling of vitamin D. Vitamin D is obtained through dietary sources, fortified food, supplements, and UVB-induced cutaneous synthesis. Following hepatic conversion to 25-hydroxyvitamin D [25(OH)D], the major circulating form, it undergoes further hydroxylation in the kidneys to generate the biologically active metabolite 1,25-dihydroxyvitamin D [1,25(OH)_2_D], which exerts its effects through binding to the VDR.

Regardless of its source, vitamin D undergoes hydroxylation in the liver to form 25-hydroxyvitamin D [25(OH)D], the principal circulating biomarker of vitamin D status. A second hydroxylation, primarily in the kidneys, generates the biologically active metabolite 1,25-dihydroxyvitamin D [1,25(OH)_2_D] (calcitriol), which exerts its biological effects through binding to the vitamin D receptor (VDR) [15,30]. The sources, metabolism, and biological activation of vitamin D are summarized in Figure 1. The VDR is widely expressed in numerous tissues, including intestinal epithelial and immune cells, where it regulates genes involved in immune responses, epithelial barrier integrity, and intestinal homeostasis [31,32,33].

Beyond its classical role in calcium and phosphorus homeostasis, vitamin D exerts important immunomodulatory and anti-inflammatory effects through VDR-mediated signaling [15,34]. It regulates innate and adaptive immune responses, suppresses the production of pro-inflammatory cytokines, and modulates key signaling pathways involved in inflammation, including NF-κB and JAK/STAT [11,18]. In the intestine, vitamin D contributes to the maintenance of epithelial barrier integrity by regulating intercellular junctions, supporting mucosal repair, and preserving intestinal homeostasis [31,32,33]. Furthermore, vitamin D enhances antimicrobial and antiviral defence mechanisms, thereby contributing to host resistance against infectious agents [11,33].

Vitamin D deficiency is a widespread global health problem associated with impaired immune regulation, disruption of epithelial barrier function, and chronic inflammation [10,35,36]. Clinically, vitamin D deficiency is generally defined as a serum 25(OH)D concentration below 50 nmol/L (<20 ng/mL) [37]. In addition to its well-established effects on skeletal health, accumulating evidence indicates that vitamin D deficiency also contributes to systemic immune and inflammatory dysregulation [10,35]. Increasing evidence suggests that these alterations may promote the development of gastrointestinal disorders through impaired VDR signaling, immune dysregulation, and loss of intestinal barrier integrity [31,32,33]. These observations provide the biological rationale for examining the role of vitamin D in intestinal diseases discussed in the following sections.

## 4. Alternative Natural Sources and Industrial Synthesis of Vitamin D

Natural dietary sources of vitamin D_3_ (cholecalciferol) include fatty marine fish, fish liver oil, liver, and egg yolk, with fatty fish representing one of the richest natural sources of vitamin D [16,38]. However, dietary intake alone is often insufficient to maintain adequate vitamin D status, particularly in individuals with dietary restrictions or impaired absorption [30,36].

In addition to endogenous cutaneous synthesis and animal-derived foods, several non-animal sources of vitamin D have been identified. Mushrooms exposed to ultraviolet light produce vitamin D_2_ through photochemical conversion of ergosterol [39,40], whereas microalgae have emerged as promising alternative sources of vitamin D_3_ for plant-based and vegan formulations [41]. Although higher plants are generally considered poor sources of vitamin D, vitamin D-related compounds have also been identified in several plant species [42].

**Figure 2:**
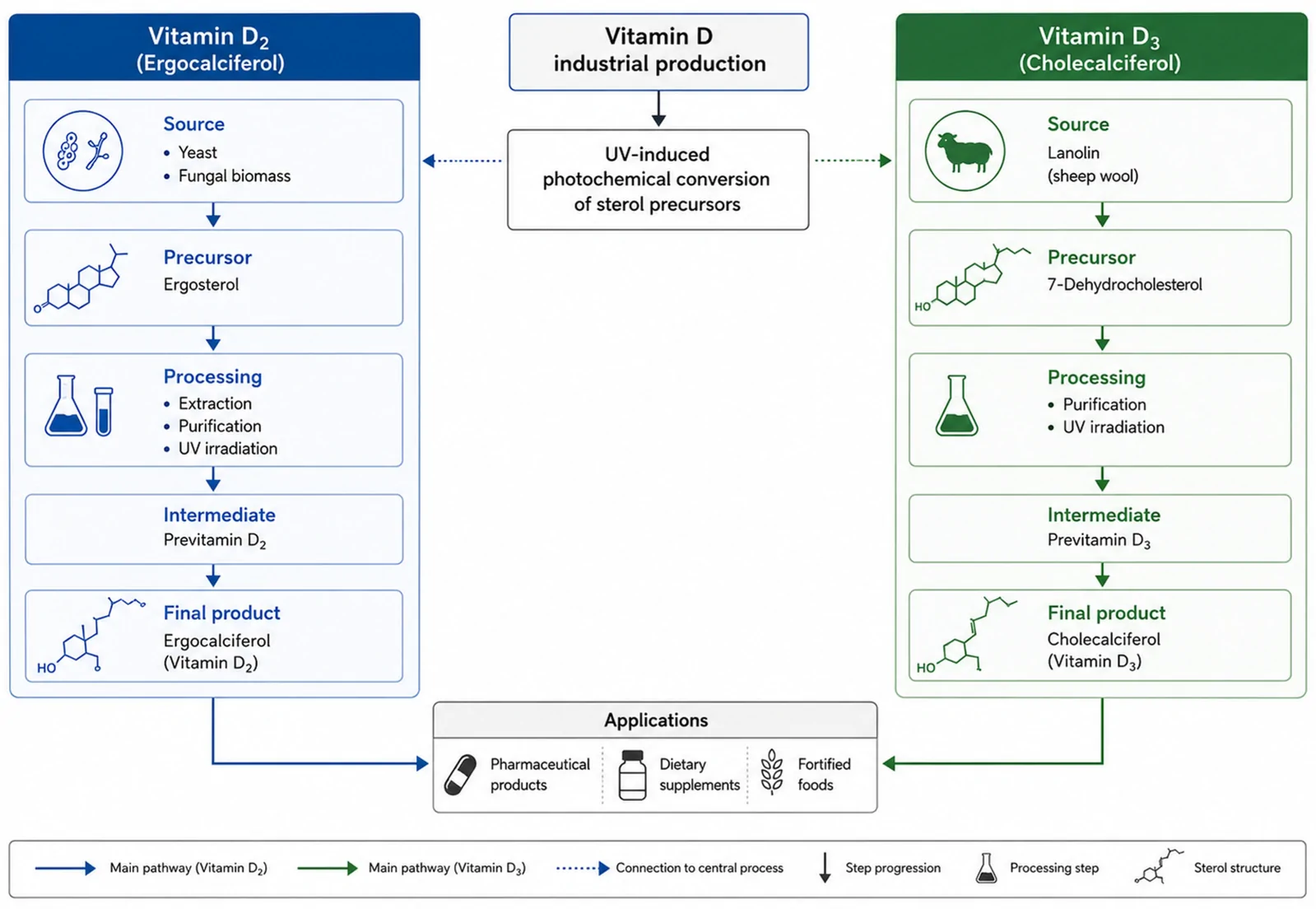
Schematic overview of the industrial production of vitamin D_2_ and vitamin D_3_. Vitamin D_2_ is synthesized from ergosterol derived from yeast or fungal biomass, whereas vitamin D_3_ is synthesized from 7-dehydrocholesterol obtained from lanolin. Both compounds undergo ultraviolet-induced photochemical conversion followed by purification before use in pharmaceutical products, dietary supplements, and fortified foods.

Because natural sources alone are often insufficient to ensure adequate vitamin D intake, industrial production remains essential for manufacturing pharmaceutical preparations, dietary supplements, and fortified foods. Vitamin D_2_ is synthesized from ergosterol derived from yeast or fungal biomass, whereas vitamin D_3_ is synthesized from 7-dehydrocholesterol obtained from lanolin. Both compounds undergo ultraviolet-induced photochemical conversion followed by purification before use in commercial products. An overview of industrial vitamin D production is presented in Figure 2 [30,40].

## 5. Role of Vitamin D in Immune Regulation: Innate and Adaptive Immunity, Anti-Inflammatory Effects, and Molecular Mechanisms

The intestinal immune system maintains a delicate balance between immune tolerance toward dietary antigens and the commensal microbiota and effective defence against invading pathogens [43,44]. This highly specialized immune network comprises gut-associated lymphoid tissue (GALT), the intestinal epithelium, lamina propria, and mesenteric lymph nodes, which collectively coordinate local immune responses and preserve intestinal homeostasis [43]. Continuous communication between epithelial cells, immune cells, and the gut microbiota maintains epithelial barrier integrity and prevents excessive inflammatory responses under physiological conditions [45,46]. Disruption of this tightly regulated balance contributes to chronic intestinal inflammation and the development of immune-mediated intestinal diseases [43,47].

The intestinal immune microenvironment contains resident and recruited innate and adaptive immune cell populations that cooperate to maintain immune surveillance, antimicrobial defence, immune tolerance, and mucosal homeostasis (Table 1) [48,49,50]. Most intestinal immune cells originate from bone marrow-derived precursors and subsequently acquire tissue-specific phenotypes under the influence of the intestinal microenvironment [51]. During inflammation or infection, additional leukocyte populations are recruited to the intestinal mucosa through chemokine- and cytokine-dependent signalling pathways to promote pathogen clearance, tissue repair, and resolution of inflammation [52,53]. However, excessive or persistent leukocyte recruitment may exacerbate tissue injury and promote chronic inflammatory responses [54].

In pathological conditions such as inflammatory bowel disease, disruption of intestinal immune homeostasis is characterised by excessive recruitment and activation of inflammatory immune cells, increased production of pro-inflammatory cytokines, and impaired regulatory immune mechanisms. Persistent activation of Th1- and Th17-mediated immune responses, together with reduced regulatory T-cell activity, contributes to sustained production of inflammatory mediators and chronic intestinal inflammation. These immune abnormalities are accompanied by dysfunction of macrophages and dendritic cells, altered innate lymphoid cell activity, epithelial barrier disruption, and dysbiosis, resulting in continuous crosstalk between immune activation and epithelial injury [61,64].

Consequently, restoration of immune homeostasis has become a major therapeutic objective, and therapeutic strategies aimed at restoring immune tolerance while preserving antimicrobial defence have emerged as promising approaches for the management of inflammatory bowel disease [64].

VDR is expressed in key immune cells, including T and B lymphocytes, monocytes, macrophages, and dendritic cells, highlighting the role of vitamin D in immune regulation [11,65]. An important feature is the ability of immune cells to express the enzyme 1α-hydroxylase, which enables the local conversion of 25(OH)D into its biologically active form, 1,25(OH)_2_D [66]. Local production of the active vitamin D metabolite allows it to act in autocrine and paracrine ways directly within the immune microenvironment, regulating cell proliferation, differentiation, and functional activity. This mechanism helps regulate the immune response while limiting excessive inflammatory activation, forming the molecular basis for the immunomodulatory effects of vitamin D [67].

Vitamin D plays an important role in regulating innate immunity by promoting the differentiation of monocytes and macrophages and enhancing their ability to respond to pathogens [68,69]. It also promotes the induction of antimicrobial peptides, including cathelicidin (LL-37) and β-defensins, thereby strengthening innate antimicrobial defence [70,71]. In addition, vitamin D modulates the functional activity of innate immune cells by regulating toll-like receptor signalling, reducing the production of pro-inflammatory cytokines, and increasing IL-10 synthesis, thereby limiting excessive inflammatory responses and maintaining immune homeostasis [72]. These effects are mediated through activation of the VDR, which regulates the transcription of genes involved in immune responses [71]. Reduced vitamin D levels are associated with decreased LL-37 production and increased susceptibility to infections, highlighting its importance in innate host defence [71,72]. Vitamin D also contributes to macrophage phagocytic activity and maintenance of epithelial barrier function, further supporting mucosal immune homeostasis [35,73,74].

**Figure 3:**
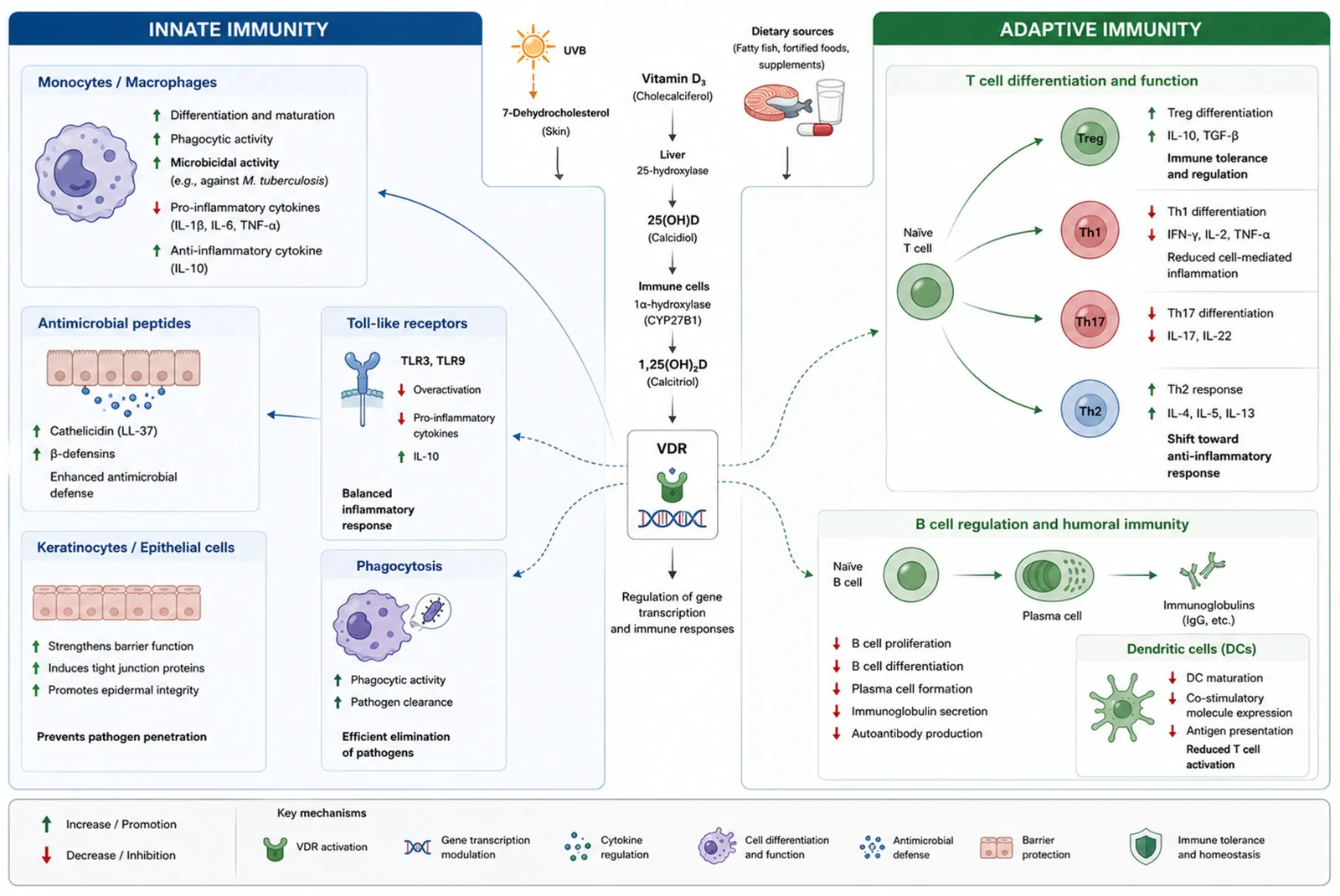
Immunomodulatory effects of vitamin D on innate and adaptive immunity. Vitamin D regulates innate immune responses through modulation of macrophage function, antimicrobial peptide production, toll-like receptor signaling, and epithelial barrier integrity. Its effects on adaptive immunity include suppression of Th1/Th17-mediated responses, promotion of Treg activity and Th2 polarization, and regulation of B-cell proliferation, plasma cell differentiation, and immunoglobulin production through VDR-dependent signalling pathways.

Vitamin D promotes an anti-inflammatory adaptive immune profile by stimulating the activity of regulatory T cells and suppressing T helper 1 (Th1)- and T helper 17 (Th17)-mediated immune responses [75]. One of the key effects of vitamin D is reducing the Th17/Treg ratio, which is associated with limiting autoimmune inflammation and maintaining immune tolerance [76]. Furthermore, vitamin D promotes a shift in the adaptive immune response from a pro-inflammatory Th1 profile towards Th2-mediated responses while limiting the production of pro-inflammatory cytokines [35]. In addition to its effects on T cells, vitamin D is involved in the regulation of humoral immunity by influencing B-cell function and autoantibody production [75]. The immunomodulatory effects of vitamin D are also linked to its ability to suppress the proliferation and differentiation of T and B lymphocytes, reduce immunoglobulin secretion and plasma cell differentiation, limit the immunostimulatory activity of dendritic cells, and induce apoptosis in activated B cells, collectively reducing the severity of adaptive inflammatory responses (Figure 3) [77,78,79,80,81].

Recent studies show that the anti-inflammatory effects of vitamin D are associated with the ability to induce the polarization of macrophages and microglia towards the anti-inflammatory M2 phenotype, as well as to suppress the release of pro-inflammatory cytokines through VDR-dependent signalling pathways [82,83]. Clinical data suggest that vitamin D deficiency is associated with increased levels of pro-inflammatory cytokines, including IL-6, IL-8, IL-33, and IFN-γ, thereby contributing to an enhanced inflammatory response [84]. Experimental and clinical studies demonstrate that vitamin D supplementation helps reduce the production of pro-inflammatory cytokines, including IL-6, as well as the severity of inflammatory responses [85]. Furthermore, vitamin D can reduce IL-17 levels and the Th17/Treg ratio, thereby helping restore immune homeostasis and limit autoimmune inflammation [76]. An important mechanism underlying its anti-inflammatory action is the stimulation of IL-10 production, which has strong immunoregulatory properties and helps maintain immune homeostasis [75].

The molecular effects of vitamin D are primarily mediated through activation of the VDR, which, upon binding to its ligand, forms a complex with the retinoid X receptor and regulates the expression of genes involved in immune responses, inflammation, and cellular homeostasis [86,87,88]. VDR-dependent regulation involves chromatin remodelling, transcriptional activation, and recruitment of co-regulatory complexes, thereby enabling fine-tuning of cellular signalling pathways [86,89]. Recent studies indicate that vitamin D acts not only through transcriptional regulation but also through modulation of translational processes and the mTOR signalling pathway, which contributes to the control of cellular activity and inflammatory responses [90]. VDR-mediated mechanisms also involve the Sirt1/PGC1α signalling axis, which is associated with the regulation of cellular metabolism, oxidative stress, and cellular ageing [91]. An important mechanism underlying the anti-inflammatory effects of vitamin D is suppression of the NF-κB signalling pathway, accompanied by reduced expression of pro-inflammatory mediators, including IL-1β and TNF-α [92]. In addition, vitamin D modulates the PI3K/Akt and MAPK/ERK signalling pathways, which are associated with reduced expression of IL-6 and TNF-α and suppression of inflammatory and apoptotic processes [93]. The immunomodulatory effects of vitamin D are also linked to epigenetic regulation of NF-κB-dependent signalling through modification of histone acetylation and suppression of RelB transcriptional activity [94]. Together, these VDR-mediated transcriptional, epigenetic, and signalling mechanisms underline the key role of vitamin D in regulating immune responses, inflammation, and cellular homeostasis.

Vitamin D has strong antiviral and immunomodulatory properties, helping to limit viral replication, reduce the expression of viral entry receptors, and regulate inflammatory responses during viral infections [69,95]. Its immunomodulatory effects in viral infections are linked to reduced production of pro-inflammatory cytokines, including TNF-α, IL-6, and IFN-γ, as well as regulation of inflammation-associated microRNAs [96]. Furthermore, vitamin D can enhance the early antiviral response by modulating transcriptional programs associated with interferon-stimulated genes and the antiviral activity of innate immune cells [97]. Its antiviral effects also involve the induction of antimicrobial peptides, including β-defensins, as well as modulation of toll-like receptor-dependent mechanisms of innate immunity, which help limit viral replication and reduce the severity of inflammatory responses [98]. Another mechanism underlying the antiviral effects of vitamin D is the regulation of metabolic signalling pathways, including AKT-dependent mechanisms involved in cellular activation and viral replication [99].

Disruptions in VDR-mediated immune regulation and vitamin D deficiency are considered important contributors to immune dysregulation, increased susceptibility to infectious diseases, and autoimmune conditions [100]. Disruption of immune homeostasis associated with vitamin D deficiency is also considered an important pathogenic factor in inflammatory bowel disease and chronic gastrointestinal mucosal inflammation [70,101,102]. Given the key role of vitamin D in regulating innate and adaptive immune responses, its deficiency may contribute to persistent chronic inflammation, impaired immune tolerance, and the development of inflammatory bowel disease.

## 6. Role of Vitamin D in Gut Barrier Function: Epithelial Integrity, Mucosal Defence, and Microbiota Interactions

The intestinal barrier is a highly coordinated, multilayered defence system comprising physical, chemical, immunological, and microbial components that collectively protect the host against luminal pathogens and other harmful substances while maintaining intestinal homeostasis (Table 2) [103,104,105]. Accumulating evidence indicates that vitamin D and its receptor (VDR) play important roles in regulating both the structural and functional integrity of the intestinal barrier, thereby influencing mucosal immune defence and host–microbiota interactions [106,107,108,109].

The physical component of the intestinal barrier is formed by the intestinal epithelium, a single-cell layer that creates a structural boundary between the intestinal lumen and the internal environment of the host [110,111]. This highly organised barrier comprises enterocytes, goblet cells, Paneth cells, enteroendocrine cells, and specialised M cells involved in the transport of luminal antigens to mucosal immune cells [109,111]. Tight junctions, composed of proteins including ZO-1, claudins, and occludin, represent the principal structural element of the epithelial barrier by regulating paracellular permeability and maintaining epithelial integrity [103,104,112,113].

**Table 2:** Effects of Vitamin D/VDR Signaling on Major Components of the Intestinal Barrier.

Component of the Intestinal Barrier	Role in Intestinal Barrier Function	Effects of Vitamin D/VDR Signalling	Consequences of Vitamin D Deficiency for Barrier Function	References
Tight junctions	Regulation of paracellular permeability; maintenance of epithelial barrier integrity; preservation of intercellular adhesion between epithelial cells	Regulation of tight junction protein expression (claudins, ZO-1, occludin); maintenance of epithelial barrier integrity; reduction of paracellular permeability	Disrupted tight junction protein expression; increased intestinal permeability (“leaky gut”); impaired epithelial barrier function; enhanced translocation of microorganisms and antigens	[105,106,108,112,114]
Mucus layer	First line of physical and chemical defence; limitation of microbial and toxin contact with the epithelium; physical separation of the epithelial surface from luminal content	Maintenance of goblet cell function; promotion of mucin production; preservation of mucus layer integrity; support of mucosal barrier function	Goblet cell dysfunction; weakened mucosal barrier; reduced antimicrobial defence; increased microbial contact with the epithelium	[106,108,111,113]
Paneth cells	Secretion of antimicrobial peptides (α-defensins); prevention of bacterial infiltration; participation in innate intestinal immune defence; regulation of inflammatory responses	Maintenance of Paneth cell function; support of innate antimicrobial defence; preservation of intestinal immune homeostasis	Paneth cell dysfunction; impaired innate antimicrobial defence; disruption of intestinal homeostasis; increased susceptibility to inflammation	[5,101,107]
Gut microbiota	Colonisation resistance; competitive exclusion of pathogens; production of antimicrobial substances; maintenance of intestinal homeostasis; regulation of mucosal barrier function	Modulation of gut microbiota composition and diversity; support of microbial homeostasis; maintenance of host–microbiota interactions	Microbial dysbiosis; reduced colonisation resistance; increased growth of opportunistic microorganisms	[5,105,115,116,117]
Mucosal immune barrier	Local mucosal immune defence; limitation of pathogenic microorganism proliferation; immune surveillance against pathogens	Modulation of local immune responses; maintenance of mucosal immune homeostasis; support of immune surveillance	Impaired local immune responses; weakened mucosal immune defence; increased intestinal inflammatory activity	[105,106,118,119]

Intestinal stem cells, located at the base of the intestinal crypts, are responsible for continuous epithelial renewal and regeneration following injury [120,121]. They generate the major epithelial cell lineages, thereby preserving the structural and functional integrity of the intestinal mucosa under physiological conditions and restoring epithelial continuity after injury through coordinated proliferation, differentiation, and migration along the crypt–villus axis [120,122].

In pathological conditions such as inflammatory bowel disease, chronic inflammation, oxidative stress, microbial dysbiosis, and cytokine-mediated tissue damage may disrupt ISC function and impair epithelial regeneration [123]. Dysregulated ISC activity can contribute to defective mucosal healing, persistent barrier dysfunction, and prolonged intestinal inflammation [94]. Conversely, effective epithelial repair requires coordinated ISC activation, controlled differentiation, and restoration of the epithelial barrier [124]. Therefore, ISC-mediated regeneration represents a central mechanism linking epithelial homeostasis, mucosal healing, and disease recovery in inflammatory intestinal disorders [123,125].

Vitamin D/VDR signalling has been implicated in the regulation of epithelial integrity and mucosal repair [12,33]. Experimental evidence suggests that vitamin D may support intestinal epithelial regeneration by promoting epithelial cell survival, regulating proliferation and differentiation, and enhancing restitution after injury [33,126]. In particular, vitamin D/VDR-dependent mechanisms have been associated with improved epithelial repair responses, including promotion of epithelial regeneration and restoration of mucosal integrity following injury [33].

The chemical component of the intestinal barrier is formed by the mucus layer, which serves as the first line of physical and chemical defence by trapping microorganisms and toxins and preventing their direct contact with the epithelial surface [115,127]. The structural basis of this barrier consists of mucins produced by goblet cells, among which MUC2 is the principal component of the mucus layer, limiting microbial contact with and adhesion to the epithelium [105]. Additional chemical protection is provided by antimicrobial molecules, including defensins and other antimicrobial peptides, which inhibit the growth of pathogenic microorganisms and limit their contact with the epithelial surface [109,110,128].

The biological component of the intestinal barrier is formed by the commensal gut microbiota, which helps protect the host by limiting the growth of pathogenic microorganisms through mechanisms of competitive exclusion, the production of antimicrobial substances, and the establishment of colonisation resistance [106,115,116]. The commensal microbiota also plays an important role in maintaining intestinal homeostasis by interacting with the mucosal barrier and influencing goblet cell differentiation and mucin maturation [116,117].

The immunological component of the intestinal barrier is mediated by local immune defence mechanisms, in which secretory immunoglobulin A plays a key role by limiting the colonisation and proliferation of pathogenic microorganisms on the mucosal surface [105,109,110]. Cells of the gut-associated lymphoid tissue also contribute significantly to intestinal immune defence by providing immune surveillance and supporting mucosal defence mechanisms [118,119].

The structural integrity of the intestinal epithelial barrier is largely determined by specialised intercellular junctions that mediate cell adhesion, selective permeability, and controlled transport across the epithelial layer [5]. Tight junctions play a central role by regulating paracellular transport and limiting the uncontrolled penetration of ions, solutes, microbial components, and other luminal factors. These dynamic multiprotein complexes comprise transmembrane proteins (occludin, claudins, and junctional adhesion molecules) and cytoplasmic zonula occludens proteins (ZO-1, ZO-2, and ZO-3), which anchor them to the actin cytoskeleton [5,96,101,103,104]. Claudins primarily determine paracellular permeability, whereas occludin contributes to tight junction stability and epithelial barrier function [129]. Junctional adhesion molecules also participate in inflammatory regulation and immune cell migration, while adherens junctions and desmosomes provide additional mechanical stability to the epithelial layer [112,130]. Disruption of tight junctions by inflammatory mediators, oxidative stress, or microbial toxins increases intestinal permeability, promotes translocation of luminal antigens, and activates inflammatory responses [5].

The VDR is widely expressed throughout the gastrointestinal tract, including enterocytes, crypt epithelial cells, and epithelial cells of both the small and large intestines, highlighting the direct role of vitamin D in maintaining barrier function and intestinal homeostasis [106,107,108,131]. VDR signalling regulates intestinal epithelial cell proliferation, differentiation, and apoptosis, thereby preserving mucosal integrity and epithelial function. Furthermore, vitamin D promotes mucosal regeneration and epithelial restitution following injury, which is particularly important during inflammatory and infectious intestinal damage [131,132]. VDR activation also supports the expression of intercellular junction proteins, including occludin, zonula occludens proteins (ZO-1), and claudins, thereby preserving tight junction integrity and regulating paracellular transport [42,107,131,133]. Conversely, vitamin D deficiency impairs VDR signalling, reduces junction protein expression, disrupts barrier integrity, and increases intestinal permeability, facilitating the translocation of luminal antigens and microbial components [131,133]. In addition, vitamin D protects the intestinal epithelium by suppressing NF-κB signalling and reducing pro-inflammatory cytokine production, thereby maintaining epithelial integrity under inflammatory stress [67,107].

Whilst damage to tight junctions reflects a structural defect in the barrier system, increased intestinal permeability (‘leaky gut’) represents its functional consequence, characterised by the excessive passage of luminal components through the intestinal epithelium [134,135]. When barrier integrity is compromised, bacterial components, lipopolysaccharides, endotoxins, food antigens, and microbial metabolites can penetrate the epithelial layer and translocate into the lamina propria, where they activate innate and adaptive immune responses. This process promotes the production of pro-inflammatory cytokines, mucosal injury, and persistent inflammation, creating a pathological cycle that further impairs barrier function and drives intestinal inflammatory damage [5,8]. In this context, vitamin D deficiency contributes to disruption of epithelial intercellular junctions, increased intestinal permeability, and exacerbation of mucosal inflammation [131,133].

In addition to regulating intercellular junctions and maintaining epithelial integrity, vitamin D contributes to intestinal mucosal defence by modulating innate immune mechanisms and promoting mucosal repair. VDR-dependent signalling preserves mucosal barrier function by regulating mucin production, maintaining mucus layer integrity, and supporting goblet cell function, thereby limiting direct contact of luminal microorganisms and toxins with the epithelial surface [132]. Vitamin D also enhances local innate antimicrobial defence through the induction of antimicrobial peptides, including LL-37 and β-defensins, which limit pathogen colonisation and support local immune homeostasis [136,137]. Furthermore, it helps maintain Paneth cell function, thereby contributing to the production of antimicrobial molecules and preservation of intestinal microbial homeostasis [5]. In addition, vitamin D promotes mucosal healing and intestinal epithelial regeneration during inflammatory or infectious stress [131,132]. Thus, the protective effects of vitamin D extend beyond tight junction stabilisation to encompass antimicrobial defence, mucosal immune homeostasis, and epithelial repair.

## 7. Role of Vitamin D in Intestinal Diseases: Pathophysiological Mechanisms and Clinical Evidence

Given the pleiotropic effects of vitamin D, including its immunomodulatory actions, maintenance of intestinal epithelial barrier integrity, and influence on host–microbiota interactions, its potential role in the pathogenesis and clinical course of various intestinal diseases has attracted considerable scientific interest. Vitamin D deficiency is considered a potential contributing factor to the development of chronic inflammation, increased intestinal permeability, immune dysregulation, and alterations in gut microbiota composition, all of which may influence the onset and progression of various intestinal disorders [12]. The most substantial body of evidence has been accumulated to date in relation to inflammatory bowel diseases, including Crohn’s disease (CD) and ulcerative colitis (UC); however, the potential role of vitamin D is also being investigated in other intestinal disorders, including irritable bowel syndrome, coeliac disease, intestinal infections, and colorectal cancer [12,13,138]. An overview of the currently available clinical evidence regarding vitamin D supplementation in major intestinal diseases, including study design, supplementation regimen, treatment duration, clinical outcomes, and study limitations, is presented in Table 3.

Overall, the clinical evidence summarized in Table 3 indicates that the therapeutic effects of vitamin D differ considerably across intestinal diseases. The most consistent evidence has been reported for inflammatory bowel diseases, particularly Crohn’s disease, where vitamin D supplementation has been associated with reduced risk of clinical relapse and improved maintenance of remission in selected patient populations. By contrast, evidence for ulcerative colitis remains less robust, with pooled analyses demonstrating inconsistent effects on disease activity despite encouraging findings from individual studies. Similarly, although vitamin D supplementation may reduce symptom severity in irritable bowel syndrome, its effects on quality of life remain uncertain. For infectious diarrhoeal diseases, coeliac disease, and colorectal cancer, current evidence is limited and does not yet support routine clinical use of vitamin D supplementation as a disease-specific therapeutic intervention [139,140,141,142,143].

The heterogeneity of clinical findings observed across studies evaluating vitamin D supplementation in intestinal diseases is likely attributable to both biological differences among disease entities and substantial methodological variability between published studies. These discrepancies may reflect differences in disease pathogenesis, the role of vitamin D in disease development, and variations in study design and patient populations [144,145].

Baseline vitamin D status represents another important source of variability. Even when identical cholecalciferol supplementation regimens are used, changes in serum 25(OH)D concentrations may differ considerably between individuals, and analyses based solely on average treatment effects may obscure clinically relevant interindividual variability. Baseline serum 25(OH)D concentration may therefore serve as a potential predictor of individual responsiveness to vitamin D supplementation [145,146]. However, existing evidence primarily demonstrates its influence on changes in circulating 25(OH)D concentrations following supplementation, whereas its impact on clinically meaningful outcomes in intestinal diseases remains insufficiently established [146].

Additional sources of heterogeneity include differences in vitamin D formulations, supplementation regimens and dosages, treatment duration, concomitant medications, and patient characteristics such as age, comorbidities, and nutritional status. Furthermore, variations in study design, sample size, duration of follow-up, clinical endpoints, and outcome assessment limit direct comparison between studies and reduce the certainty of pooled estimates. Collectively, these factors underscore the need for standardized, adequately powered randomized controlled trials evaluating not only changes in serum 25(OH)D concentrations but also clinically meaningful outcomes to better define the therapeutic role of vitamin D in intestinal diseases [145].

Future large-scale, adequately powered randomized controlled trials are needed to determine the optimal vitamin D formulations, dosing regimens, treatment duration, and target patient populations. Such studies will also help define the translational potential of vitamin D as an adjunctive strategy for preserving intestinal barrier function, modulating gut immunity, and improving clinically relevant outcomes in intestinal diseases [147].

CD is a chronic, relapsing inflammatory bowel disease whose pathogenesis is closely linked to dysregulation of innate intestinal immunity, altered host–microbiota interactions, and pathological activation of Th1- and Th17-mediated immune responses [148,149]. Numerous studies, including meta-analyses, consistently demonstrate an association between CD and vitamin D deficiency; furthermore, reduced serum 25(OH)D levels are associated with higher disease activity [148,149,150]. Vitamin D deficiency in patients with CD may result from malabsorption due to small bowel involvement, insufficient dietary intake, and limited sunlight exposure, as well as alterations in vitamin D metabolism and transport [117]. Of particular interest is the involvement of vitamin D in regulating molecular mechanisms directly implicated in the pathogenesis of CD. The VDR has been shown to interact with CD susceptibility genes, including NOD2 and ATG16L1, which play key roles in bacterial recognition, autophagy, and innate intestinal antimicrobial defence [148,149]. Disruption of VDR-dependent signalling may contribute to immune dysregulation, reduced mucosal antimicrobial defence, impaired Paneth cell function, and alterations in gut microbiota composition, all of which may support the persistence of chronic intestinal inflammation in CD [148].

UC is a chronic inflammatory bowel disease characterised by continuous, diffuse inflammation of the colonic mucosa; its development is associated with an abnormal immune response, genetic predisposition, oxidative stress and altered interactions with the gut microbiota [151]. The potential role of vitamin D in the pathogenesis of UC is due to its involvement in regulating the immune response, modulating inflammatory cytokines, maintaining immune homeostasis and preserving the integrity of the intestinal epithelial barrier [86,151,152,153]. It has been shown that 1,25(OH)_2_D_3_/VDR signalling helps maintain the differentiated state of colonic epithelial cells, whereas its disruption may compromise the structure of the intestinal epithelial barrier and contribute to the development of disease flares [152,153]. Disruption of VDR-dependent signalling may be accompanied by increased inflammatory activity, damage to the intestinal mucosa, impaired tight junction function, dysregulation of immune homeostasis, and progression of the inflammatory process [86,151,152,153]. Thus, vitamin D can be regarded as a potentially significant modifiable factor capable of influencing the course of UC by maintaining the epithelial barrier, immune homeostasis, and mucosal defence [86,152,153].

IBS is a disorder of gut–brain interaction whose pathogenesis is multifactorial and involves abnormalities in intestinal permeability, immune regulation, visceral hypersensitivity, alterations in gut microbiota composition, psychosocial factors, and dysregulation of the gut–brain axis [154,155]. Accumulating evidence also indicates a high prevalence of vitamin D deficiency among patients with IBS, with an inverse association suggested between serum vitamin D levels and symptom severity [154,156,157]. In recent years, vitamin D has been considered a potentially important modifiable factor in IBS due to its ability to influence multiple pathogenic mechanisms. It has been suggested that vitamin D may help reduce dysbiosis, maintain intestinal barrier function, enhance antimicrobial peptide production, and modulate innate and adaptive immune responses. Furthermore, vitamin D is involved in regulating T-cell responses by inhibiting Th1- and Th17-mediated inflammation and promoting the induction of regulatory T cells, which may help reduce inflammatory activity and support immune tolerance in the gut. Given the association between inflammation, visceral hypersensitivity, and abdominal pain perception, these mechanisms may partly explain the influence of vitamin D on IBS symptom severity [154]. Vitamin D may also affect patients’ quality of life; however, despite encouraging findings, the available clinical evidence remains limited and does not permit definitive conclusions regarding the therapeutic efficacy of vitamin D supplementation in IBS [157,158].

Intestinal infections constitute an important group of conditions whose development and severity depend not only on pathogen virulence, but also on the effectiveness of innate mucosal defence, the integrity of the intestinal epithelial barrier, and the coordination of immune responses [138,159]. Vitamin D is considered an important regulator of host defence against infection due to its ability to enhance innate immunity and stimulate the production of antimicrobial peptides, including cathelicidin. Furthermore, vitamin D/VDR signalling is involved in the regulation of inflammatory signalling pathways and mucosal immune defence, which may help limit bacterial invasion and intestinal epithelial damage [159]. Vitamin D/VDR signalling may also contribute to the regulation of autophagy, an important mechanism of cellular antimicrobial defence that facilitates the elimination of intracellular pathogens [160]. Given these mechanisms, vitamin D deficiency may be associated with increased susceptibility to intestinal infections and a more severe disease course [138,159,160].

Celiac disease is a chronic immune-mediated disorder of the small intestine, triggered by gluten exposure in genetically predisposed individuals and characterised by inflammatory damage to the intestinal mucosa, impaired barrier function, and nutrient malabsorption [161,162]. Accumulating evidence also indicates a higher prevalence of vitamin D deficiency in patients with celiac disease, including paediatric populations [140,163]. Vitamin D deficiency in patients with celiac disease may arise both from impaired small intestinal absorption caused by inflammatory mucosal damage and villous atrophy, and from nutritional restrictions; moreover, such deficiencies may persist or develop even during long-term adherence to a gluten-free diet [161,162,164,165,166]. The pathogenesis of celiac disease involves activation of innate and adaptive immune responses, predominantly Th1-mediated inflammation, release of pro-inflammatory cytokines, disruption of intercellular junction integrity, including tight junction dysfunction, and increased intestinal permeability [161,162]. Given the immunomodulatory and barrier-protective properties of vitamin D, including its ability to modulate T-cell responses towards a more tolerogenic phenotype, vitamin D deficiency may contribute to the persistence of inflammation and worsening of disease course, although the clinical efficacy of routine vitamin D supplementation in celiac disease remains inconclusive [162,165].

Colorectal cancer is one of the most common malignancies of the gastrointestinal tract, and its development is influenced by a combination of genetic, immunological, and environmental factors [167]. In recent years, increasing evidence has suggested a potential role of vitamin D in the prevention and progression of colorectal cancer [168]. Low circulating concentrations of 25-hydroxyvitamin D [25(OH)D] have repeatedly been associated with an increased risk of colorectal cancer, poorer prognosis, and reduced overall survival [169]. These effects are thought to be mediated through activation of the vitamin D receptor (VDR), which regulates epithelial cell proliferation and differentiation, promotes apoptosis, suppresses inflammatory signalling pathways, and may inhibit activation of the Wnt/β-catenin pathway, a key driver of colorectal carcinogenesis [167]. Despite compelling experimental evidence, clinical findings remain inconsistent [170]. Observational studies have generally demonstrated an inverse association between vitamin D status and colorectal cancer risk, whereas randomized clinical trials have not consistently confirmed reductions in disease incidence or improvements in clinical outcomes following vitamin D supplementation. This heterogeneity may be attributable to differences in baseline vitamin D status, vitamin D formulations and dosages, treatment duration, study populations, and disease stage [167,170]. Overall, current evidence suggests that vitamin D may represent a promising adjunctive strategy in colorectal cancer prevention and management; however, further adequately powered randomized clinical trials are required before routine clinical implementation can be recommended [170].

## 8. Vitamin D and the Gut Microbiota: Mechanistic Interactions and Therapeutic Implications

The gut microbiota constitutes a complex and dynamic microbial ecosystem that exists in constant interaction with the host and is characterised by taxonomic, functional, and biochemical interrelationships that play a key role in maintaining intestinal homeostasis. The gut microbiome is often regarded as a functional ‘microbial organ’ involved in nutrient metabolism, the production of biologically active metabolites, including short-chain fatty acids (SCFAs), and the maintenance of colonisation resistance by limiting the growth of pathogenic microorganisms. An important mechanism through which the gut microbiota influences the host is the production and modification of microbial metabolites, including bile acids and other signalling molecules, which participate in host–microbiota communication and contribute to the maintenance of intestinal homeostasis [171,172]. Alterations in the composition and functional activity of the gut microbiota (dysbiosis), accompanied by taxonomic, biochemical, and metabolic shifts, are considered important contributors to the development of various intestinal diseases [173].

**Figure 4:**
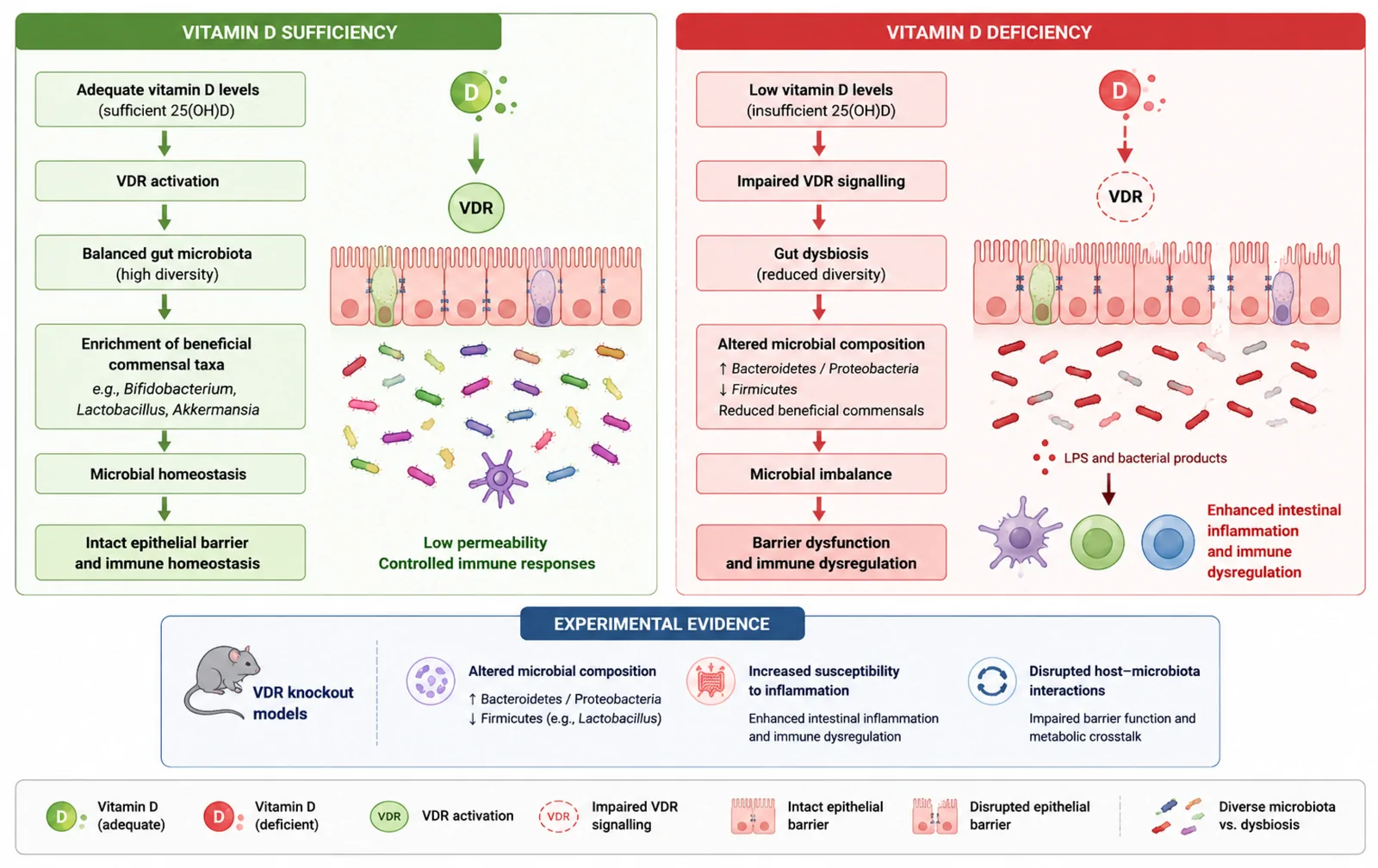
Impact of vitamin D/VDR signalling on gut microbiota composition in intestinal homeostasis and dysbiosis. Adequate vitamin D status promotes VDR activation, supports microbial diversity, and gut microbial homeostasis, whereas vitamin D deficiency is associated with dysbiosis, altered microbial composition, and disrupted host–microbiota interactions. Evidence from VDR-knockout models further highlights the role of VDR signalling in maintaining microbial homeostasis.

Vitamin D/VDR signalling plays an important role in regulating the composition of the gut microbiota. Available evidence suggests that vitamin D can modulate the composition and diversity of the gut microbiome, whereas its deficiency is associated with dysbiotic alterations and disruption of microbial homeostasis [174,175]. Furthermore, changes in VDR signalling, including genetic variations in the vitamin D receptor, are considered potential factors influencing gut microbiota composition. Studies in VDR-knockout models have demonstrated marked alterations in microbial composition, including an increase in the relative abundance of Bacteroidetes and Proteobacteria alongside a decrease in Firmicutes compared with wild-type animals, further underscoring the important role of VDR in maintaining microbial homeostasis [176]. Clinical data also indicate an association between vitamin D supplementation and an increased abundance of certain potentially beneficial bacterial taxa, including Bifidobacterium, whereas vitamin D deficiency and impaired VDR signalling are associated with reductions in specific commensal microorganisms and dysbiotic alterations in gut microbiota composition [174,175,176,177]. Importantly, the interaction between vitamin D and the gut microbiota appears to be bidirectional: vitamin D influences microbiome composition, whilst the gut microbiota, in turn, may modulate vitamin D metabolism (Figure 4) [178].

The gut microbiota and vitamin D/VDR signalling closely interact in the regulation of intestinal immune homeostasis [179,180]. The gut microbiota plays a fundamental role in immune education by influencing the development, maturation, and functional differentiation of both innate and adaptive immune cells [47]. Available evidence suggests that vitamin D/VDR signalling can modulate the intestinal immune response, including the stimulation of Treg-mediated anti-inflammatory mechanisms, and can also influence gut microbiota composition and the formation of the mucosal immune microenvironment [179,180]. In turn, the gut microbiota, through changes in the microbial environment and the production of metabolites, including SCFAs, may contribute to the regulation of intestinal immune responses and the maintenance of immune tolerance, including through effects on regulatory T-cell differentiation [180,181]. Microbial signalling molecules, including pathogen-associated molecular patterns and short-chain fatty acids, act as important mediators of immune communication between the gut microbiota and the host, influencing intestinal epithelial signalling, antimicrobial defence, and immune cell differentiation [181]. In addition, the interaction between vitamin D and the gut microbiota appears to be bidirectional, as the gut microbiota may modulate vitamin D metabolism and the expression of its receptor, forming a complex regulatory network linking the microbial community, inflammatory processes, and the intestinal immune system. Taken together, these findings suggest a functional overlap between microbiota-mediated immune signalling pathways and vitamin D/VDR-dependent mechanisms involved in the regulation of intestinal immune homeostasis [179,180,181].

Microbial metabolites are considered key chemical mediators in communication between the gut microbiota and the host, transmitting signals that can influence metabolic, immune, and epithelial processes. In this context, vitamin D/VDR signalling represents an important regulator of the interaction between the gut microbiota and the host metabolome, as VDR is involved in shaping gut microbiota composition and related metabolic profiles. Disruption of VDR signalling has been associated with dysbiotic changes, including a reduction in butyrate-producing bacteria, as well as marked alterations in carbohydrate, lipid, amino acid, and bile acid metabolism [182]. Among microbial metabolites, SCFAs, including butyrate, acetate, and propionate, are of particular interest, as they are produced through bacterial fermentation in the colon. These metabolites have immunomodulatory properties and contribute to the regulation of anti-inflammatory processes and the maintenance of intestinal homeostasis [183]. In addition to SCFAs, secondary bile acids also play an important role in microbiota-mediated signalling and may interact with vitamin D-dependent pathways. In particular, lithocholic acid, a microbial metabolite, is considered an agonist of the vitamin D receptor, suggesting a direct molecular interaction between microbial metabolites and VDR signalling. Furthermore, changes in microbial metabolite profiles, including reduced levels of secondary bile acids, may be associated with impaired vitamin D metabolism and bioavailability [171]. These observations suggest that microbial metabolites may participate in the regulation of immunometabolic processes linking the metabolic activity of the gut microbiota with immune and epithelial homeostasis [182,183,184].

One of the most obvious therapeutic approaches for modulating the vitamin D–gut microbiota axis is the correction of vitamin D status through supplementation. However, interpretation of clinical data is complicated by the lack of consistency in the forms of vitamin D used, as the term “vitamin D supplementation” may refer to cholecalciferol, ergocalciferol, calcidiol, or calcitriol in different studies. These forms differ in their biological properties, stability, and physiological roles, which should be considered when evaluating therapeutic efficacy [185]. Additional evidence suggests that vitamin D supplementation may modulate gut microbiota composition, potentially contributing to anti-inflammatory effects and metabolic homeostasis [186]. At the same time, probiotics and prebiotics are considered promising tools for therapeutic modulation of the gut microbiota because of their ability to influence microbial composition, barrier function, and mucosal immune mechanisms. Proposed mechanisms include strengthening the mucosal barrier, increasing the expression of tight junction proteins, promoting the growth of beneficial microbial communities, and reducing inflammatory activity. Combined strategies, including the use of vitamin D together with probiotics or prebiotics, as well as synbiotic approaches, are of particular interest, as they may provide additive or synergistic effects in correcting intestinal dysbiosis and restoring intestinal homeostasis [180,187]. In addition, increasing attention is being given to microbiome-targeted therapeutic strategies aimed at more precise modulation of microbial composition, microbial metabolism, and related immunometabolic signalling pathways, in line with the concept of personalised therapy for intestinal diseases [6].

## 9. Future Perspectives

Despite significant progress in understanding the role of vitamin D in regulating gut immunity, maintaining barrier function, and interacting with the gut microbiota, several important unresolved questions remain in this field. Although accumulated experimental and clinical data suggest the potential importance of the vitamin D–gut microbiota axis in the pathogenesis of intestinal diseases, the results of clinical trials evaluating the efficacy of vitamin D supplementation remain inconclusive [188,189]. Such variability may reflect not only the marked heterogeneity of study designs, differences in study populations, disease phenotypes, intervention duration, and the forms and dosages of vitamin D used, but also the fundamental biological complexity of the host–microbiota interaction system itself. Additional confounding factors may also contribute, including patients’ baseline vitamin D status, dietary patterns, sun exposure levels, geographical differences, antibiotic use, body mass index, and concomitant therapies, all of which complicate the interpretation of clinical findings and limit their reproducibility [176,188].

An additional methodological limitation is that a substantial proportion of the available data is based on observational studies or small-scale experimental models, while the causal mechanisms underlying the interaction between vitamin D, the gut microbiota, and clinical outcomes in humans remain poorly understood [176,190]. In particular, it remains unclear whether vitamin D deficiency is a direct pathogenic factor contributing to the development of gut dysfunction, or whether, in some cases, it reflects a secondary consequence of chronic inflammation, malabsorption, or broader metabolic dysfunction. Similarly, distinguishing the direct effects of vitamin D supplementation from secondary changes mediated by alterations in the gut microbiota, immune responses, or metabolic signalling pathways remains challenging. Interpretation of the available data is further complicated by the lack of standardisation in microbiome analysis methods, including differences in sample collection, sequencing, bioinformatic processing, and taxonomic resolution, which complicates comparison across studies and limits the ability to draw reproducible conclusions.

Another important challenge is the marked inter-individual variability in the composition and functional activity of the gut microbiota, which may contribute to differences in response to vitamin D supplementation and other microbiome-targeted interventions [14,191]. This raises an important conceptual question about whether a standardised therapeutic approach is appropriate in a system characterised by high biological variability. It is likely that the clinical response to vitamin D is determined not only by baseline vitamin D status, but also by the specific microbial profile, the functional metabolic capacity of the microbiota, and the state of the intestinal barrier. The high clinical heterogeneity of intestinal diseases also warrants further attention, as the potential effects of vitamin D may vary depending on the specific disease, the stage of the pathological process, and the degree of inflammatory activity [12].

Given the complexity of interactions between vitamin D, the gut microbiota, microbial metabolites, the immune system, and intestinal barrier function, future research should go beyond the assessment of isolated clinical outcomes and adopt a more integrated analysis of host–microbiota interactions. Large-scale randomised controlled trials with standardised intervention protocols will be of particular importance; however, even such studies may prove insufficient on their own without adequate biological stratification of patients. In this regard, the integration of microbiome, metabolome, and immunological profiling appears critical for a more accurate understanding of the mechanisms underlying therapeutic responses. Another important area is the conduct of translational human studies aimed at more precisely characterising the molecular mechanisms linking VDR signalling, microbial metabolites, and immune pathways in clinical settings.

In the long term, one of the most promising directions is a shift from universal supplementation-based approaches to personalised therapeutic strategies based on individual microbiota characteristics, immune status, metabolic profile, and baseline vitamin D status. Such a precision medicine approach may facilitate more accurate selection of patients who are most likely to derive clinical benefit from targeted modulation of the vitamin D–gut microbiota axis [14,176].

## 10. Conclusions

This review highlights the multifaceted role of vitamin D in maintaining intestinal homeostasis through its coordinated effects on immune regulation, epithelial barrier integrity, and gut microbiota composition. Beyond its classical role in calcium and phosphorus metabolism, vitamin D modulates innate and adaptive immune responses, promotes antimicrobial peptide production, preserves epithelial barrier function, supports mucosal repair, and regulates host–microbiota interactions through vitamin D receptor (VDR)-dependent signaling pathways. Collectively, these mechanisms provide a biologically plausible basis for the involvement of vitamin D in the pathogenesis of inflammatory bowel disease, irritable bowel syndrome, celiac disease, intestinal infections, and colorectal cancer.

Current evidence indicates that vitamin D deficiency is frequently associated with increased disease activity, impaired epithelial barrier function, immune dysregulation, and alterations in gut microbiota composition. However, whether vitamin D deficiency represents a causal factor in the development of intestinal diseases or is primarily a consequence of chronic inflammation, impaired nutrient absorption, and disease progression remains uncertain. Likewise, although vitamin D supplementation has demonstrated beneficial effects in experimental studies and selected clinical trials, the available evidence is insufficient to support its universal therapeutic use across all intestinal diseases.

The inconsistency of the available evidence reflects substantial methodological and clinical heterogeneity across published studies rather than uniformly contradictory findings. Clinical investigations differ considerably with respect to patient populations, disease phenotypes and severity, baseline vitamin D status, vitamin D formulations and dosages, treatment duration, outcome measures, and study design. Furthermore, a considerable proportion of the current evidence is derived from observational studies or experimental models, limiting causal inference and direct translation into clinical practice. These limitations emphasize the need for cautious interpretation of existing findings and highlight the importance of further high-quality clinical research.

Future studies should focus on well-designed randomized controlled trials using standardized vitamin D supplementation protocols, clinically relevant outcome measures, and appropriate patient stratification according to baseline vitamin D status, disease phenotype, and gut microbiota characteristics. Integrating immunological, microbiome, metabolomic, and molecular data will improve our understanding of the mechanisms underlying vitamin D action and help identify patient populations most likely to benefit from targeted interventions. Such an integrated precision medicine approach may contribute to the development of more effective strategies for the prevention and management of intestinal diseases. Although current evidence supports an important biological role of vitamin D in intestinal homeostasis, its routine therapeutic use should await confirmation from adequately powered randomized clinical trials.

## Figures and Tables

**Table 1:** Major immune cell populations involved in intestinal immune homeostasis and their regulation by vitamin D.

Immune Cell Population	Physiological Role	Changes in Intestinal Diseases	Effect of Vitamin D	References
Macrophages	Phagocytosis, clearance of pathogens and apoptotic cells, maintenance of intestinal homeostasis	Increased M1 polarization, excessive cytokine production	Promotes M2 polarization, suppresses TNF-α, IL-6, IL-1β	[55,56]
Dendritic cells	Antigen presentation, induction of immune tolerance	Enhanced activation, promotes Th1/Th17 responses	Induces tolerogenic phenotype, reduces antigen presentation	[50,55]
Innate lymphoid cells (ILCs)	Early mucosal defence, IL-22 production, epithelial repair	Dysregulated cytokine production	Supports ILC3-derived IL-22 production and epithelial barrier homeostasis	[57,58]
Neutrophils	Rapid antimicrobial defence	Excessive infiltration and tissue injury	Limits excessive neutrophil infiltration and inflammatory activation	[55,59]
Regulatory T cells	Maintenance of immune tolerance	Reduced number/function	Promotes Treg differentiation and immunosuppressive function	[55,60]
Th17 cells	Protection against extracellular pathogens	Excessive activation, IL-17 production	Suppresses Th17 differentiation and reduces IL-17 production	[55,61]
B cells/plasma cells	IgA production, humoral immunity	Enhanced B-cell activation and dysregulated antibody responses	Inhibits B-cell proliferation, plasma-cell differentiation, and immunoglobulin production	[55,62]
Intestinal epithelial cells	Barrier integrity, antimicrobial peptide production, epithelial regeneration	Barrier disruption, impaired regeneration	Enhances tight junction integrity, antimicrobial peptide expression, and epithelial regeneration	[46,63]

**Table 3:** Summary of key evidence on vitamin D status and clinical relevance across intestinal diseases.

Disease	Evidence Type	Vitamin D Formulation	Vitamin D Regimen	Treatment Duration	Main Findings	Overall Evidence	Clinical Implications	Limitations	References
Crohn’s disease	Systematic review and meta-analysis of 12 randomized controlled trials (RCTs)	Predominantly oral cholecalciferol (vitamin D3)	250–10,000 IU/day or equivalent weekly/intermittent regimens	4–52 weeks	Vitamin D supplementation reduced the risk of clinical relapse, with the strongest effect observed in CD patients in clinical remission (RR 0.47; 95% CI 0.27–0.82). Improvement in disease activity scores was inconsistent	Moderate-certainty evidence supports reduced relapse risk in patients with Crohn’s disease in remission; evidence for improving disease activity remains inconsistent	Vitamin D supplementation may be considered as an adjunctive strategy for maintaining remission, particularly in CD patients with vitamin D deficiency	Small sample sizes, heterogeneous supplementation regimens, and low-to-moderate certainty of evidence	[139]
Ulcerative colitis	Systematic review and meta-analysis of 12 RCTs	Predominantly oral cholecalciferol (vitamin D3)	500–60,000 IU/day or equivalent weekly/intermittent regimens	4–52 weeks	Vitamin D supplementation showed potential benefits in individual studies; however, pooled analyses did not demonstrate a significant improvement in clinical disease activity in UC patients	Current evidence is insufficient to support routine vitamin D supplementation for disease control	Vitamin D supplementation may be considered as an adjunctive therapy in patients with vitamin D deficiency; however, current evidence is insufficient to support routine use for disease control	Limited UC-specific RCTs, heterogeneous interventions, and low-certainty evidence	[139]
Irritable bowel syndrome	Systematic review and meta-analysis of 8 clinical studies (685 patients)	Predominantly oral cholecalciferol (vitamin D3)	2000–50,000 IU/day or equivalent weekly regimens	6–24 weeks	Vitamin D supplementation was associated with a significant reduction in IBS symptom severity (IBS-SSS), but no statistically significant improvement in quality of life (IBS-QoL)	Moderate evidence supports improvement in symptom severity, whereas benefits for quality of life remain uncertain	Vitamin D supplementation may provide symptomatic benefit in selected patients, particularly those with vitamin D deficiency	High heterogeneity, variable supplementation regimens, and limited IBS subtype stratification	[140]
Diarrhoeal diseases	Systematic review of 4 clinical studies (including 1 RCT)	Predominantly oral cholecalciferol (vitamin D3)	Single oral dose or daily supplementation (study-dependent)	Variable (study-dependent)	Current evidence does not support a significant benefit of adjunctive vitamin D3 supplementation in childhood infectious diarrhoea. Overall evidence remains insufficient	Current evidence does not support routine adjunctive vitamin D supplementation in childhood infectious diarrhoea	Routine adjunctive vitamin D supplementation cannot currently be recommended for the management of childhood infectious diarrhoea	Limited clinical evidence, only one RCT, high heterogeneity, and insufficient data for meta-analysis	[141]
Coeliac disease	Meta-analysis of 27 studies (28 datasets; 1137 patients and 2613 controls)	—	—	—	Patients with coeliac disease had significantly lower serum 25(OH)D levels than controls (WMD −8.36 nmol/L; 95% CI −14.63 to −2.09). Serum 25(OH)D levels normalized following a gluten-free diet. No significant association with VDR polymorphisms was observed	Vitamin D deficiency is common and should be corrected; however, evidence for therapeutic benefits beyond correction remains limited	Assessment and correction of vitamin D deficiency may be considered as part of long-term disease management	Predominantly observational evidence, high heterogeneity, and lack of prospective and randomized controlled studies	[142]
Colorectal cancer	Systematic review and meta-analysis of RCTs	Predominantly oral cholecalciferol (vitamin D3)	Variable across included trials	Variable across included trials	Vitamin D supplementation was associated with improved overall survival in patients with colorectal cancer; however, evidence regarding disease-free survival and progression remained limited	Evidence suggests improved overall survival, but larger high-quality RCTs are required before routine clinical recommendations can be made	Vitamin D supplementation may represent a promising adjunctive strategy in colorectal cancer management, although further adequately powered RCTs are required	Limited number of RCTs, heterogeneous study populations and supplementation regimens, and moderate certainty of evidence	[143]

## Data Availability

No new data were created or analyzed in this study. Data sharing is not applicable to this article.

## Abbreviations

The following abbreviations are used in this manuscript:

VDR	vitamin D receptor
SCFAs	Short-chain fatty acids
CD	Crohn’s disease
UC	ulcerative colitis
IBS	irritable bowel syndrome
LL-37	cathelicidin
Th1	T helper type 1 cells
Th17	T helper type 17 cells
Treg	regulatory T cells
IL	interleukin
ZO-1	zonula occludens-1
TNF-α	tumor necrosis factor alpha
NF-κB	nuclear factor kappa B
MUC2	mucin 2
RCTs	randomized controlled trials
